# American Medical Society

**Published:** 1853-11

**Authors:** 


*American Medical Association.*

At a meeting of the American Medical Association, held at New York, in May 1853, the undersigned were appointed to receive voluntary communications on medical subjects, and to award two prizes of $100 each, to the authors of the two best essays.

Each communication must be accompanied by a sealed packet, containing the name of the author, which will be opened only in the case of the successful competitors.

Unsuccessful communications will be returned on application after June 1st, 1854.

Communications must be addressed, post-paid, to the Chairman of the Committee, Dr. Charles A. Pope, 123 Locust street, St Louis, Missouri, on or before the 30th of March, 1854.

Chas. A. Pope, M. D.
Thomas Reyburn, M. D.
John S. Moore, M. D.
John B. Johnson, M. D.
A. Litton, M. D.
*Committee.*
Chas. A. Pope, M. D.
*Chairman.*

*"Old Wine in new bottles."*

Dr. Dixon in the last number of his racy journal, the *Scalpel*, charges us with a very grave crime. We published in our Sept. No., an article headed "*Great capacity for Physic.*" The article was taken from the *Kentucky Medical Recorder*, we found it there without credit, but as it was among selections from other Journals, we supposed that it was not original with our confreres of the *Recorder*. Illegitimacy is our horror, and we always advocated the doctrine that every child should have a daddy. We confess, however, to an obtuseness in not seeing a relation between the pungency of the article and the keenness of the Scalpel.

We had nearly forgotten that very lucid argument, in Plato's Gorgias, we think it is, where it is shown conclusively, that every cut must be such a cut as the thing cutting cuts. J.

